# Rapid learning curve assessment in an ex vivo training system for microincisional glaucoma surgery

**DOI:** 10.1038/s41598-017-01815-z

**Published:** 2017-05-09

**Authors:** Yalong Dang, Susannah Waxman, Chao Wang, Hardik A. Parikh, Igor I. Bussel, Ralitsa T. Loewen, Xiaobo Xia, Kira L. Lathrop, Richard A. Bilonick, Nils A. Loewen

**Affiliations:** 1 Department of Ophthalmology, School of Medicine, University of Pittsburgh, Pittsburgh, Pennsylvania United States; 2 The Third Xiangya Hospital of Central South University, Changsha, Hunan China; 3 Xiangya School of Medicine, Central South University, Changsha, Hunan China; 4 Department of Ophthalmology, Xiangya Hospital of Central South University, Changsha, Hunan China

## Abstract

Increasing prevalence and cost of glaucoma have increased the demand for surgeons well trained in newer, microincisional surgery. These procedures occur in a highly confined space, making them difficult to learn by observation or assistance alone as is currently done. We hypothesized that our *ex vivo* outflow model is sensitive enough to allow computing individual learning curves to quantify progress and refine techniques. Seven trainees performed nine trabectome-mediated ab interno trabeculectomies in pig eyes (n = 63). An expert surgeon rated the procedure using an Operating Room Score (ORS). The extent of outflow beds accessed was measured with canalograms. Data was fitted using mixed effect models. ORS reached a half-maximum on an asymptote after only 2.5 eyes. Surgical time decreased by 1.4 minutes per eye in a linear fashion. The ablation arc followed an asymptotic function with a half-maximum inflection point after 5.3 eyes. Canalograms revealed that this progress did not correlate well with improvement in outflow, suggesting instead that about 30 eyes are needed for true mastery. This inexpensive pig eye model provides a safe and effective microsurgical training model and allows objective quantification of outcomes for the first time.

## Introduction

Microincisional glaucoma surgeries (MIGS)^1, 2^ are performed in a submillimeter-wide space approximately 200-fold smaller than that available for traditional epibulbar glaucoma drainage devices^3^. This restriction makes these techniques difficult to master even for experienced anterior segment surgeons. There is potential for injury to the iris root, ciliary body band, suprachoroidal space, outer wall of Schlemm’s canal, and the vessels contained in those structures. The trabecular meshwork can be easily confused with anatomic landmarks in close proximity that look similar such as Schwalbe’s line or the ciliary body band. Consequences are often minor but may occasionally be severe^4, 5^. Surgical success depends on navigating the intricacies of visualizing the angle, identifying the correct target, avoiding trauma, and maximizing the ablation length. With experience, surgeons can expand their indications from primary open angle glaucomas to eyes that are relatively contraindicated, for instance after failed trabeculectomy^6^ or tube shunts^7^, eyes with narrow angles^8^, and eyes with peripheral anterior synechiae^6, 9^.

Synthetic models or simulators similar to those available for cataract surgery^10^ do not exist for angle surgery and may not replicate the subtle tactile feedback that is characteristic of the trabecular meshwork and Schlemm’s canal. Although human donor eyes could be used in theory, priority must be given to tissue suitable for transplantation. Additionally, the combination of storage time, corneal quality, and postmortem corneal edema in human tissue available for practice typically prevents direct gonioscopic visualization of the anterior chamber angle^11, 12^. Exceptions have been reported within healthcare systems where tissue can be made available rapidly, but the time-limited availability complicates training and assessment^13^. As a result, most new surgeons have to learn MIGS by performing surgery in actual glaucoma patients. In this setting, surgeon trainers sitting in the assistant position cannot easily correct mishaps or take over as is readily possible in traditional epibulbar glaucoma surgery.

The positive impact of model based-training systems on surgical outcomes has been documented. The success rates of trainees can change by more than four-fold in non-ophthalmological specialties that perform minimally invasive procedures^14^. For example, the failure rate in robotic-assisted prostatectomy (cancer-free margins) improved from 45% to 12% following training on a model^15^. In cataract surgery, the rate of posterior capsular rupture can decrease approximately ten-fold to only 2% of patients following model-based training^16^.

Increasing prevalence^17^ and cost^18^ of glaucoma as a result of extended lifespan^19^ have increased the demand for well-trained MIGS surgeons. MIGS have a favorable risk profile and appropriate effectiveness for a spectrum of glaucoma types either as an initial^4, 20, 21^, or as a secondary procedure^6^, and across a range of severity^22, 23^. This suggests that MIGS surgeons may play an increasing role in reducing the economic burden of glaucoma by providing an earlier and less expensive intervention^24, 25^ than traditional glaucoma surgeries^26^.

Utilizing the leading MIGS modalities, trabectome-mediated ab interno trabeculectomy (Neomedix Inc., Tustin, CA, USA) and the iStent trabecular microbypass (Glaukos, Laguna Hills, CA, USA), we have shown that the porcine eye model enables a MIGS experience similar to that of human eyes^27–29^. Notably, this model is approximately 50 times less expensive, readily available, and allows unimpaired angle view through a clear cornea. Here, we demonstrate that not only can porcine models be used as a safe and reliable training model for ab interno trabeculectomy with the trabectome (AIT), but also that rapid feedback with objective and quantifiable data is possible. We anticipate that the availability of an accurate training model will improve patient outcomes in MIGS.

## Results

Seven trainees without experience in angle surgery participated in this study and operated on nine freshly enucleated pig eyes. No trainee had performed more than 20 phacoemulsifications with exception of trainee #3 who exceeded this number at least 10 fold.

**Figure 1:**
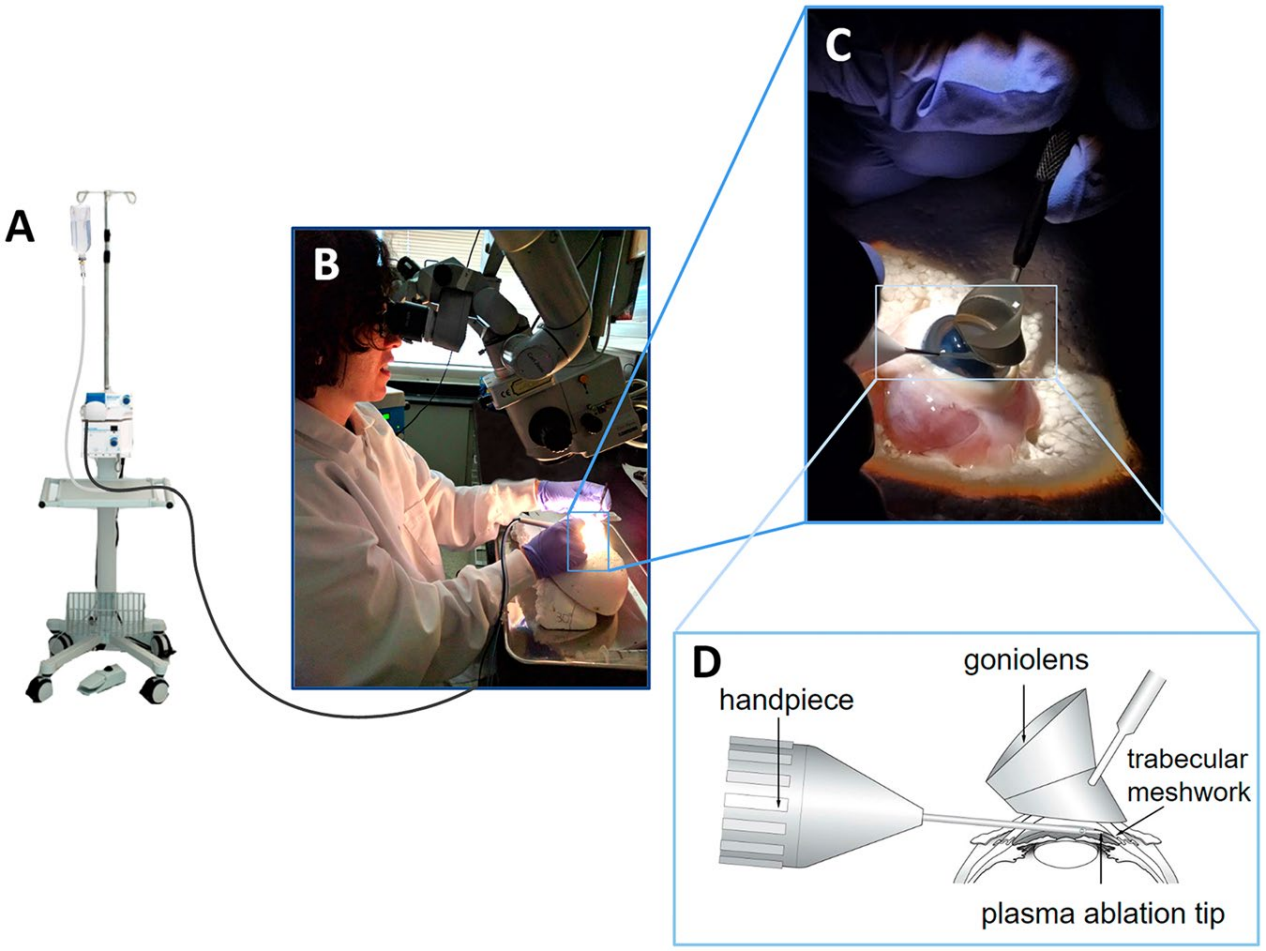
Setup for microincisional glaucoma surgery. (**A**) Trabectome stand with aspiration/irrigation pump and high frequency generator. (**B**) Trainee surgeon with rotated mannequin head and tilted ophthalmic microscope. (**C**) Right-handed trainee using a surgical goniolens with the left hand and the trabectome handpiece with the right. The trabectome is inserted through a temporal uniplanar clear corneal incision. (**D**) Under direct view, the trabecular meshwork is engaged with the tip of the trabectome and ablation is started.

The pig eye MIGS training system afforded a surgical experience that was very similar to that in human eyes. With practice, corneal incision, clear visualization of the opposing anterior chamber angle, and trabecular ablation could be accomplished (Fig. 1). Pectinate ligaments, characteristic of the porcine anterior chamber angle, provided an opportunity to practice goniosynechiolysis but could also be ablated with the trabectome tip directly. Due to the depth of the porcine TM, trainees typically had to make several ablation passes towards the left and the right until view of white sclera indicated completion. All surgeons were right-handed and followed the standard practice of first ablating counterclockwise and then clockwise.

**Figure 2:**
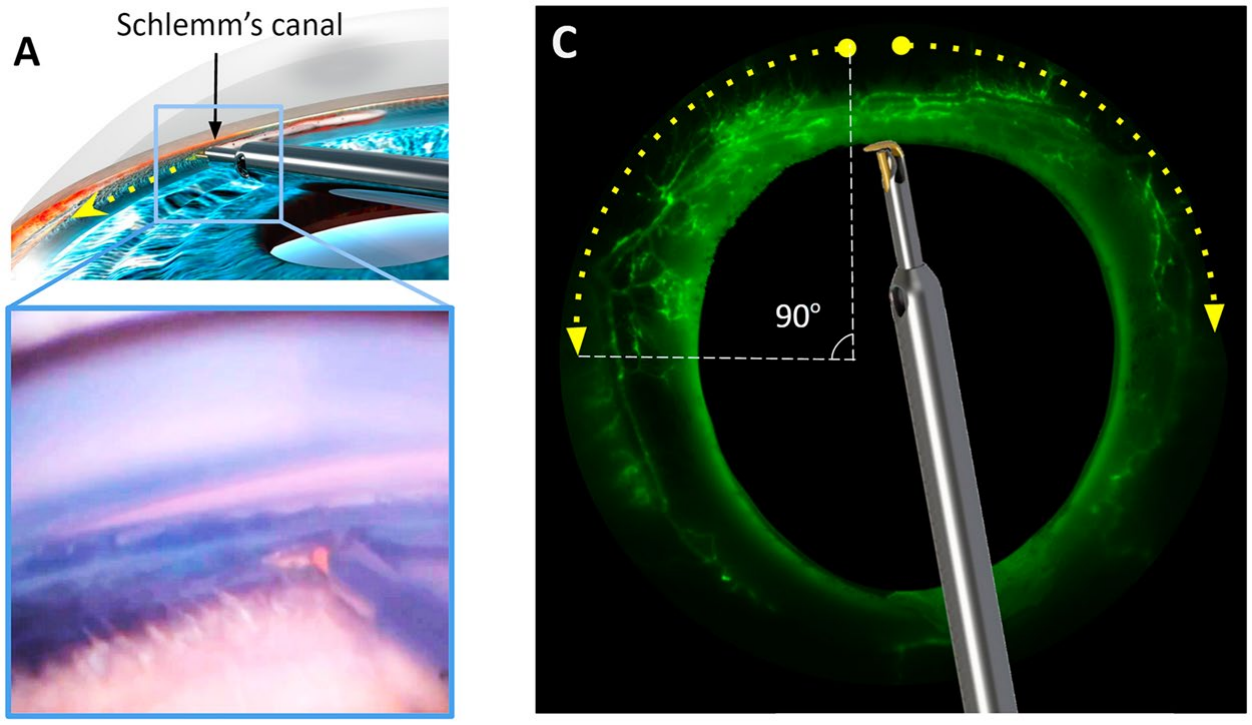
Schematic representation of the iridocorneal angle. (**A**) A microincisional glaucoma surgical device (trabectome) inserted into Schlemm’s canal in a human eye. Ablation is directed towards the left. (**B**) Direct gonioscopic view of the trabectome tip engaged in the trabecular meshwork in a pig eye. (**C**) A canalogram demonstrates fluorescent microspheres that enter the outflow tract only after successful ablation. Anterior chamber fluorescence is suppressed to avoid detraction from collector channel outflow. Up to 180 degrees (90 degrees on each side) of trabecular meshwork could be ablated as in this example.

Due to the use of fluorescent spheres, incomplete TM ablation did not lead to an increase in fluorescence, while successful ablation resulted in fluorescence throughout the connected collector channels and aqueous veins (Fig. 2), with a relatively limited spread beyond the end of the ablation arc.

### Time

Trainees became faster and more confident with each increasing eye. The relationship between time and eye number was best described with a linear mixed-effect model fit by restricted maximum likelihood (REML) and had a random intercept and random slope. Trainees became 1.4 minutes faster with each subsequent eye (P < 0.0001). The formula describing time was y = 19.88 − 1.42x.

**Figure 3:**
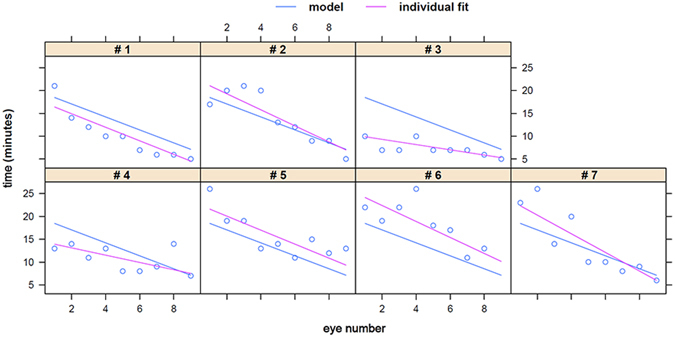
Surgical time and eye number. The relationship of eye number and time for each trainee followed a linear function.

Surgical times ranged from 25 minutes in first eyes to 5 minutes towards the end of training. The mean surgery time decreased from 21.0 ± 2.8 minutes with the first eye to 8.7 ± 1.9 minutes with the 9th eye. Surgeons who were already relatively fast experienced less improvement than surgeons who had longer operating times early on (Fig. 3).

### Arc

**Figure 4:**
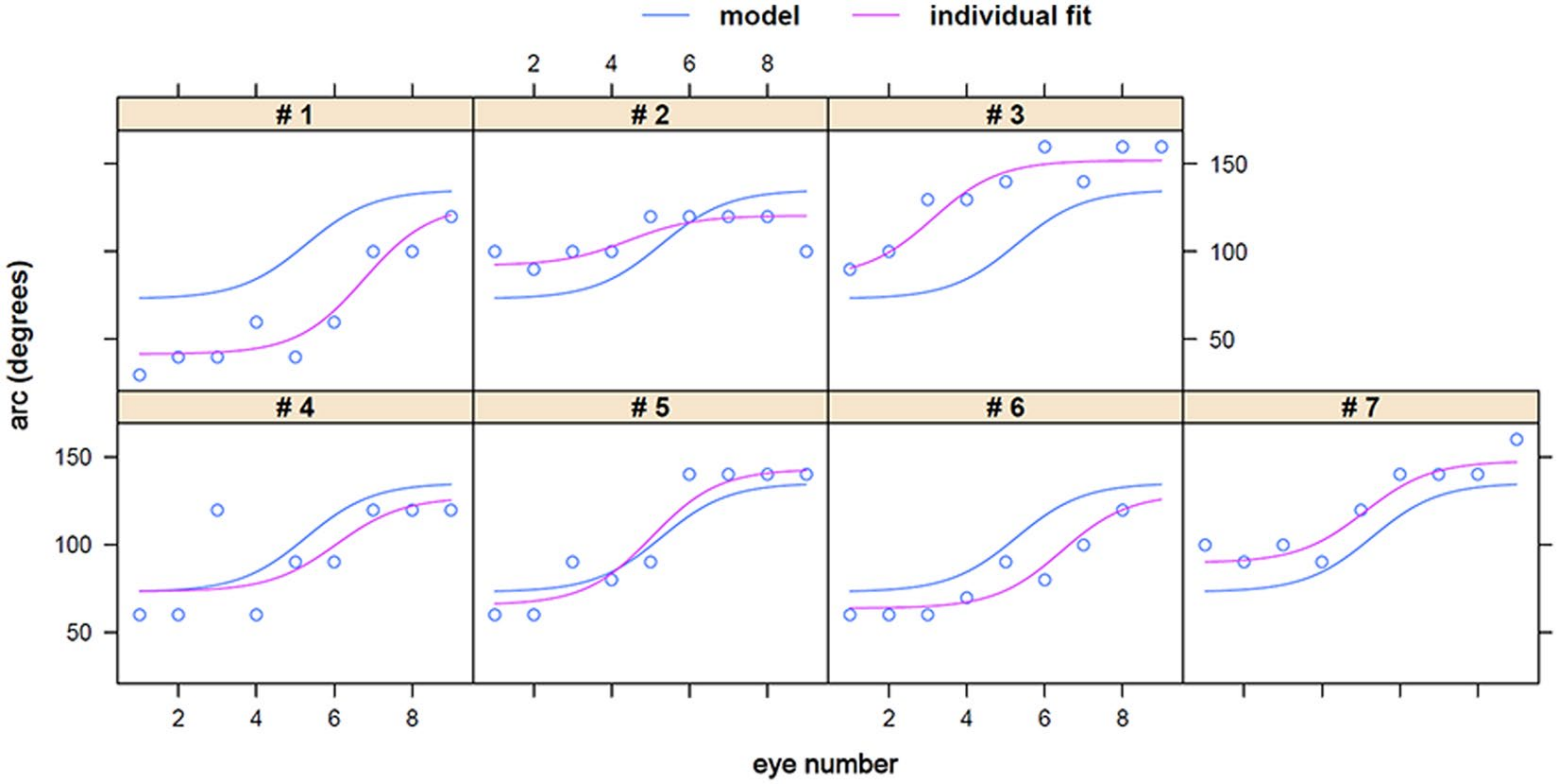
The improvement of ablation arc length could be described as a sigmoidal curve.

The ablation arc increased from a mean of 70 ± 10 degrees in the first eye to 140 ± 10 in the final eye. In contrast to time, the relationship between ablation arc length in degrees and practice eye number could be best described with an asymptotic regression with a single random effect for the asymptote ϕ1 using a nonlinear mixed-effects model approach fit by maximum likelihood, resulting in the following equation: y(x) = A + (B − A)/(1 + e^(x_mid−x)/s^) where A was the asymptote as x goes to positive infinity and B was the asymptote as x goes to negative infinity. For arc (y) as a function of eye number (x), A = 73.11, B = 135.14, x_mid = 5.27, and S = 0.85 so that y(x) = 73.11 + 62.03/(1 + e^(5.27−x)/0.85^). The parameter x_mid was the inflection point and S was the scale. The inflection point was reached at 5.27 on average (P < 0.0001), indicating that trainees had peaked in their learning speed and improvement as applied to the ablation arc length (Fig. 4).

### Operating Room Score (ORS)

**Figure 5:**
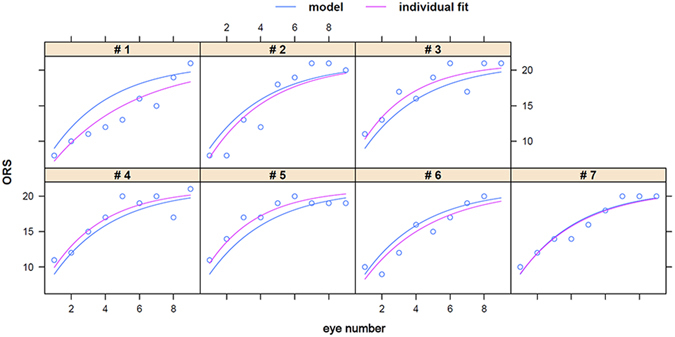
The Operating Room Score (ORS) had a hyperbolic shape that was very similar for all trainees.

The relationship between ORS and eye number was best described by an asymptotic regression model with single random effect for the asymptote ϕ1. y(x) = ϕ1 + (ϕ2 − ϕ1)e^−eϕ3 x^ where ϕ1 was the asymptote as x goes to infinity and ϕ2 was the value of y at x = 0. The parameter ϕ3 is the logarithm of the rate constant. The logarithm was used to enforce positivity of the rate constant so that the model approached an asymptote. The corresponding half-life t0.5 was: t0.5 = log2/e^ϕ3^. For ORS (y) as a function of the eye number (x), ϕ1 = 21, ϕ2 = 36.93, and ϕ3 = −1.26 so that: y(x) = 21 + 15.93e^−e(−1.26x)^ and t0.5 = 2.45. ϕ1 was fixed to 21 rather than estimated. This result indicated that trainees only required 2.45 eyes to reach half-maximal performance in this operating room compound score (P < 0.0001). The ORS range was wider at the start of the training while the final scores achieved were very similar (Fig. 5).

### Fluorescence

**Figure 6:**
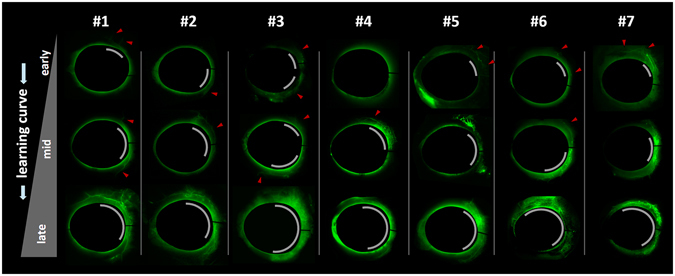
Fluorescent canalograms along the individual learning curve of trainees #1 through #7. All eyes are shown in frontal view with the superonasal quadrant in the upper right and the inferonasal, backhand ablation position in the lower right location. Microsphere canalogram casts from ablations of first eyes are visible at the top, from the middle of the learning curve in the center, and final eyes are shown at the bottom. Gray arcs indicate the extent of successful connection to the outflow tract. Most trainees initially only succeeded in ablation of the superonasal TM during the preferred, counterclockwise pass. Red arrowheads indicate aqueous veins that can be best seen in magnified view.

Canalograms (Fig. 6) were assessed by measuring the relationship between the mean increase of fluorescence and eye number as fit by REML with a random slope mixed model and without a random intercept. In this model, seven eyes were needed to reach the maximal modeled fluorescence (P < 0.022). The formula for mean fluorescence is f_mean_: y(x) = 1.132 + 0.0219x. When the assumption was made that the maximal fluorescence achieved by a single trainee could have been achieved by other trainees, the model predicted that it would take approximately 31 eyes to reach this maximal, observed level of fluorescence. The fluorescence increase could only be modeled in the superonasal quadrant, the first one encountered by right-handed surgeons, in a meaningful way. Such increased flow in this preferred quadrant was apparent in most early canalograms (Fig. 6 top, gray arcs). Eight eyes were needed to reach the maximal fluorescence predicted by the model. The formula for this quadrant was y(x) = 1.13 + 0.052y. Maximal fluorescence achieved by any of the trainees was predicted to be reached after 29 eyes.

## Discussion

In contrast to wet lab preparation for cataract surgery^30^, trainee glaucoma surgeons learn trabeculectomy and tube shunt procedures by observing and assisting a more experienced surgeon^31^. This is an acceptable practice because these procedures are performed in the subtenon or subconjunctival space on the outside of the eye and allow the senior surgeon to intervene quickly and safely; this is impossible in MIGS. To address this problem, manufacturers of MIGS devices use inverted human corneal rims for training. However, corneal rims provide only limited tactile feedback and do not allow for practicing visualization with a specialized goniolens in a pressurized eye. An inexpensive training model system would be highly desirable to prepare novice surgeons and reduce the risk of complications. Pig eyes have a relatively thick TM as well as Schlemm’s canal like segments, termed the angular aqueous plexus^32^, instead of a single lumen. They have been used to train cataract^33^ and glaucoma surgeons in goniotomy^34^, trabeculectomy^12, 35^, deep sclerectomy^36, 37^, and tube shunt implantation^38^, but without a scoring system. Fresh pig eyes obtained from an abattoir have the additional advantages of a clear cornea and absence of human pathogens.

Although it is accepted as common sense that practice before new surgeries shortens the learning curve, there is a surprising lack of objective, quantifiable evidence of this in microsurgical specialties. Of 104 studies concerned with measuring and improving surgical skills, only 7^39^ used an objective, structured assessment of technical skills (OSATS)^40^ and had a low level of evidence. We recently introduced a method that allows for quantification of focal outflow changes^27–29^ using readily available fluorescein. The present study utilizes fluorescent microspheres that can enter collector channels within the time permitted here only if the TM has been successfully ablated. In contrast to sodium fluorescein, this method provides a simple, “set-and-forget” approach to estimating the extent of drainage bed access without losing confinement to the vascular bed by diffusion into the extravascular space. This allows trainees to connect an eye to a gravity-fed, constant pressure microbead suspension infusion before proceeding with the next eye. A picture can be obtained with an epifluorescence microscope or a cobalt blue equipped camera system within a forgiving timeframe.

We assessed surgical time, ablation arc length, achieved fluorescence, and surgical operating score as parameters to gauge a trainee’s progress. Although the Operating Room Score uses the same principle as the Objective Structured Assessment of Technical Skill (OSATS) tool^40^, it is the most subjective as it depends on the assessing trainer. As reflected in the performance of trainee #3 who had performed more phacoemulsifications than the other participants, prior ophthalmic microsurgery experience has mainly an impact on the operating time but not on the other parameters assessed here. In contrast to the objective data of time, ablation arc length, and fluorescence, the Operating Room Score (ORS) also contains subjective scores. Time, arc, and ORS could be modeled using linear or nonlinear fits and indicate a relatively short learning curve. The asymptotic shape for arc and ORS suggests that, following training with this model, most surgeons will have maximized their progress well to the point where they can be confidently considered safe to operate on the eye of an actual glaucoma patient.

It was surprising that the increase in fluorescence, reflective of the amount of successful access to the outflow tract, had a considerably slower slope suggesting that true mastery can only be achieved after around 30 eyes and that the counterclockwise ablation is easier to learn. While one explanation might be that our fluorescence model does not capture progress well, this method describes fluorescence changes with a relatively high fidelity^27–29^. Instead, a potentially more troubling mechanism may be at work: both the trainee and the trainer might overestimate their performance and what constitutes surgical success. A typical real-world equivalent is how experienced cataract surgeons who are unfamiliar with angle surgery become frustrated with their first MIGS cases causing them to perceive this surgery as ineffective or even dangerous. Our model recreates this by demonstrating that even when confidence and skills have already plateaued, clinical results can lag considerably at a pace that is nearly six times slower. The presence of a more gradual, objective learning curve compared to the more optimistic, steeper but subjective curve should encourage surgeons to remain humble and committed to honing their skills. Although a physician’s overconfidence seems problematic, such positive illusions are surprisingly common. Johnson and Fowler described in a recent hallmark study^41^ that the cause is an evolutionary advantage derived from a readiness to take a controlled risk, a trait that may be a significant motivator especially in high achievers. Examples are the astonishing belief by 94% of college professors that their teaching is superior to that of their peers^42^; 88% of motor vehicle drivers think that they are better than others^43^, and 70% of students rate their leadership skills as superior^44^. Alternative explanations for our different learning curves are the Hawthorne effect, which describes the change of behavior by subjects under study simply due to their awareness of being observed^45, 46^ or the presence of a placebo effect.

Perhaps the most important aspect of this model is the ability to provide a safe learning environment with rapid feedback. Such feedback is known to produce faster learning^47^ as is the anticipation of feedback itself because it creates better learning strategies^48^. Our model allows for practice of gonioscopic visualization and TM ablation under direct view, one of the most challenging aspects of microincisional glaucoma surgery, while providing a surgical experience that is very similar to that in human eyes. It will be interesting to compare patient outcomes of trainees who prepared extensively with a pig eye model to those who did not.

## Methods

### Study Design

This study (PRO16110487) was conducted according to the Declaration of Helsinki and was designated by the Institutional Review Board of the University of Pittsburgh exempt status under section 45 CFR 46.101(b)(1) of the Code of Federal Regulations. We enrolled seven trainees without experience in angle surgery in this study and provided each with nine freshly enucleated pig eyes. Based on extensive pilot experiments involving twelve trainees with a wide range of training levels, the number of participants and pig eyes needed for this study was estimated and maximized while maintaining time and budget feasibility. Trainees carefully reviewed the didactics material on trabectome-mediated ab interno trabeculectomy (AIT) consisting of training slides (Supplemental material S1) and a movie of AIT in the pig (S1 Movie). The trainer (NAL), with a log of several thousand cases, had 9 years of experience with ab intero trabeculectomy, obtained formal trainer certification by Neomedix Inc. in 2009, and has since taught resident physicians and glaucoma fellows at academic institutions. In this study, trainees observed the trainer in this model once before completing surgery under guidance. All eyes were operated on in a continuous session for each trainee. Sufficient time existed between surgeries to provide feedback. A short break was provided if requested. The trainer rated trainees’ performance by measurement of surgical time (minutes), ablation arc (degrees), terminal canalogram fluorescence (as a quantification of outflow), and computation of an ORS as detailed below.

### Trabectome-Mediated Ab Interno Trabeculectomy in Porcine Eyes

We obtained freshly enucleated porcine eyes from a local abattoir (Thoma Meat Market, Saxonburg, PA, USA) within 2 hours of sacrifice. Because animals were not sacrificed for the purpose of doing research, the Institutional Animal Care and Use Committee (IACUC) of the University of Pittsburgh determined that no specific approval was required. All anterior chambers were cannulated with a 30-gauge needle connected to an infusion of 37 °C warm, clear Dulbecco’s Modified Eagle’s Media (DMEM, Hyclone, GE Healthcare Life Sciences, Piscataway, NJ, USA) set to 15 mmHg as used in porcine eye cultures^49^. Immediately before the procedure, eyes were placed into the orbit of a styrofoam model head tilted 30 degrees away from the surgeon with support of a wedge. AIT was performed in a fashion similar to that in human eyes^1^. The model head was positioned under a surgical microscope (S4, Carl Zeiss Meditec, Jena, Germany) such as to mimic a supine patient and with the temporal side of the eye directed toward the surgeon (Fig. 1). A 1.8 mm corneal incision was created with a keratome 2 mm anterior to the temporal limbus. The inner third of the incision was slightly flared to improve mobility and eliminate striae from torque. The model head was tilted approximately 30 degrees away from the trainee while the microscope was tilted towards the surgeon to the same extent. A goniolens (Goniolens ONT-L, #600010, Neomedix Inc., Tustin, CA, USA) was placed on the cornea to visualize the iridocorneal angle along the nasal side. Under continuous irrigation, the tip of the trabectome handpiece (Neomedix Inc., Tustin, CA, USA) was inserted into the anterior chamber. Pectinate ligaments were disinserted by gentle goniosynechiolysis^27, 28, 50^ using the smooth base plate of the device or by direct ablation. Nasal TM was engaged and ablated with a power of 1.1 mW. The ablation continued towards the left for 45 to 90 degrees, depending on the trainee’s skill level; the trabectome tip was then disengaged from the TM, rotated 180 degrees within the anterior chamber, and positioned at the initial point of ablation. Ablation continued toward the right to a similar extent. The total ablation arc was recorded. After removal of the device, the incision was sealed watertight with a drop of cyanoacrylate. The trainer supervised the entire procedure, corrected mistakes, and rated and recorded components of the Operating Room Score before answering any questions raised by the trainee.

### Operating Room Scores

Analogous to the assessment used in cataract surgery^51^, trainee performance scores consisted of global and task-specific elements. Global parameters were adopted from the Objective Structured Assessment of Technical Skill (OSATS) tool^40^, which has recently been validated through real-time scoring^52^ and more video-recorded task performance^53^. Scores were summed from the following operating room parameters: arc of ablation (≥120° = 3, 90–119° = 2, <90° = 1), procedure time (<10 minutes = 3; 10–15 minutes = 2; >15 minutes = 1), number of questions asked (0–1 question = 3; 2–5 questions = 2; >5 questions = 1), confidence of movement (fluid = 3; some hesitation = 2; awkward = 1), number of errors (none = 3; 1–2 errors = 2; ≥3 errors = 1), number of entries into the anterior chamber (2 entries = 3; 3 entries = 2; ≥4 entries = 1), and scope movement (clear economy = 3; moderate = 2; unnecessary movements = 1).

### Outflow Tract Casting with Microsphere Canalograms

The extent of outflow tract access was measured with microsphere-based canalograms immediately following each surgery. Compared to fluorescein sodium-based canalograms^28, 29^, this method is simpler and less time-dependent due to the qualities of the microspheres, with a size of 0.5 micron, (F8813, yellow-green fluorescent (505/515), Thermo Fisher Scientific, Eugene, OR) which render them unable to pass through intact trabecular meshwork within a 20 minute period^27, 28^. All canalogram results were masked of trainee identity before assessment by a single analyst (YD). Perfusate was created by diluting the microbead suspension 100-fold with 37 °C phenol red-free DMEM. A 30 gauge needle was inserted through the nasal cornea of the sealed eye 2 mm anterior to the limbus with the needle tip rested in the center of the anterior chamber and the bevel pointing up. A constant pressure infusion was carried out at the hydrostatic equivalent of 15 mmHg, the normal IOP for pigs^54^, and allowed to continue for 12 minutes. The fluorescent microsphere flow pattern was visualized with a stereo dissecting microscope (Olympus SZX16 and DP80 Monochrome/Color Camera; Olympus Corp., Center Valley, PA) equipped for fluorescent imaging (Chroma 49002 GFP cube, Chroma Technology Corp, Bellow Falls, VT). Images were acquired at baseline after the anterior chamber was filled with microsphere suspension and at 12 minutes (CellSens, Olympus Life Science, Tokyo, Japan) at a 680 × 510-pixel resolution with 16-bit depth and 2 × 2 binning at a 50 ms exposure. The outflow enhancement was quantified through measurement of fluorescence increase distal to the limbus. Fluorescence of the anterior chamber, visible through the cornea, was suppressed before image analysis as described^27–29^. Each acquired image was divided into an inferonasal (IN), superonasal (SN), superotemporal (ST), and inferotemporal (IT) quadrant. The measured areas obtained at baseline and the final time-point of canalograms were kept consistent. Raw fluorescence intensity was quantified with ImageJ (Version 1.50i, National Institute of Health)^55, 56^ by a masked observer.

### Statistics

Data was analyzed with R version 3.31^57^ (2016-06-21, nlme 3.1.128, lattice 0.20.34). We first generated lattice plots to visualize the key data including ablation arc, time, ORS, total mean fluorescence, and fluorescence by quadrant. A linear mixed effects model was applied to assess the correlation between procedure time and eye number. A nonlinear mixed effects model was applied to the ablation arc by eye number. A nonlinear mixed effects model fit by maximum likelihood was used for ORS versus eye number. Mean fluorescence and fluorescence of individual quadrants was modeled with a linear mixed effects model. The relationship was fit by restricted maximum likelihood (REML) with a random slope mixed model and without a random intercept. Approximate 95% confidence intervals were computed for all models.

### Data Availability Statement

All data generated or analysed during this study are included in this published article (and its Supplementary Information files).

## Electronic supplementary material

Supplementary Video and Table Legends

Suppl Video 1

Suppl Video 2

Supplemental Table 1
