# Supplementary material for: Rapid learning curve assessment in an ex vivo training system for microincisional glaucoma surgery
Source: Sci Rep. 2017 May 9;7:1605. doi: 10.1038/s41598-017-01815-z (PMC5431621; doi:10.1038/s41598-017-01815-z)
Supplement: Supplementary file 1 — Supplementary Video and Table Legends [file 41598_2017_1815_MOESM1_ESM.doc]

# Rapid learning curve assessment in an ex vivo training system for microincisional glaucoma surgery

Yalong Dang,^1^ Susannah Waxman,^1^ Chao Wang,^1,2,3^ Hardik A Parikh,^1^ Igor I. Bussel,^1^ Ralitsa T. Loewen,^1^ Xiaobo Xia,^4^ Kira L. Lathrop,^1^ Richard A. Bilonick,^1^ Nils A Loewen ^1#^

^1^ Department of Ophthalmology, School of Medicine, University of Pittsburgh, Pittsburgh, Pennsylvania, United States

^2^ The Third Xiangya Hospital of Central South University, Changsha, Hunan, China

^3^ Xiangya School of Medicine, Central South University, Changsha, Hunan, China

^4^ Department of Ophthalmology, Xiangya Hospital of Central South University, Changsha, Hunan, China

^#^Correspondence to loewen.nils@gmail.com

# Video 1

**Video 1.** Slide stack with technology, device and steps used in preparation for first ab interno trabeculectomy.

# Video 2

**Video 2.** Example of plasma-mediated ab interno trabeculectomy with the trabectome by an experienced surgeon.

Table 1.

**Table 1:** Outflow enhancement and Operating Room Score
